# Autophagy down regulates pro-inflammatory mediators in BV2 microglial cells and rescues both LPS and alpha-synuclein induced neuronal cell death

**DOI:** 10.1038/srep43153

**Published:** 2017-03-03

**Authors:** Claudio Bussi, Javier Maria Peralta Ramos, Daniela S. Arroyo, Emilia A. Gaviglio, Jose Ignacio Gallea, Ji Ming Wang, Maria Soledad Celej, Pablo Iribarren

**Affiliations:** 1 Centro de Investigaciones en Bioquímica Clínica e Inmunología (CIBICI-CONICET). Departamento de Bioquímica Clínica, Facultad de Ciencias Químicas, Universidad Nacional de Córdoba, Córdoba, Argentina; 2 Departamento de Química Biológica, Centro de Investigaciones en Química Biológica de Córdoba (CIQUIBIC, CONICET), Facultad de Ciencias Químicas, Universidad Nacional de Córdoba, Córdoba, Argentina; 3 Laboratory of Molecular Immunoregulation, Cancer and Inflammation Program, Center for Cancer Research, National Cancer Institute at Frederick, Frederick, Maryland 21702, USA

## Abstract

Autophagy is a fundamental cellular homeostatic mechanism, whereby cells autodigest parts of their cytoplasm for removal or turnover. Neurodegenerative disorders are associated with autophagy dysregulation, and drugs modulating autophagy have been successful in several animal models. Microglial cells are phagocytes in the central nervous system (CNS) that become activated in pathological conditions and determine the fate of other neural cells. Here, we studied the effects of autophagy on the production of pro-inflammatory molecules in microglial cells and their effects on neuronal cells. We observed that both trehalose and rapamycin activate autophagy in BV2 microglial cells and down-regulate the production of pro-inflammatory cytokines and nitric oxide (NO), in response to LPS and alpha-synuclein. Autophagy also modulated the phosphorylation of p38 and ERK1/2 MAPKs in BV2 cells, which was required for NO production. These actions of autophagy modified the impact of microglial activation on neuronal cells, leading to suppression of neurotoxicity. Our results demonstrate a novel role for autophagy in the regulation of microglial cell activation and pro-inflammatory molecule secretion, which may be important for the control of inflammatory responses in the CNS and neurotoxicity.

Autophagy is a ubiquitous eukaryotic intracellular homeostatic process affecting all cell types in multicellular organisms, whereby cells autodigest parts of their cytoplasm for removal or turnover^1^. Autophagy utilizes a conserved, eukaryotic molecular machinery that involves the sequestration of target materials and their subsequent delivery to and breakdown by the lysosome/vacuole^2^. Autophagic end-products can be released from lysosomes to enable some maintenance of the cellular energy status^3^. When environmental changes produce starvation, it starts inhibition of mammalian target of rapamycin complex 1 (mTORC1), a negative regulator of autophagy, and activation of Jun N-terminal kinase (JNK; also known as MAPK8), which induces autophagy^4^.

Neurodegenerative disorders are associated with autophagy dysregulation, and drugs modulating autophagy have been successful in several animal models. Neurodegenerative conditions, such as Alzheimer’s (AD) or Parkinson’s disease (PD), involve the accumulation of protein aggregates in neurons^5^. Since autophagy is one of the major degradative pathways that cells utilize to achieve proteostatic balance, its activation appears especially promising in potential treatment of these diseases^6^,^7^. PD is a common neurodegenerative disease characterized by the degeneration of dopaminergic neurons in the substantia nigra pars compacta (SNpc). However, the cause of PD remains elusive. Recently, emerging evidence has demonstrated that inflammatory responses manifested by glial reactions and increased expression of inflammatory cytokines are recognized as prominent features of PD. Inflammatory mediators such as nitric oxide (NO), TNFα, and interleukin-1β (IL-1β) derived from non-neuronal cells including microglia, are believed to modulate the progression of neuronal cell death in PD^8^,^9^.

Microglial cells are resident macrophages in the central nervous system (CNS)^10^ and have multiple functions, such as phagocytosis, production of growth factors and cytokines, and antigen presentation^11^. Under normal conditions, microglial cells are in a resting state, but they become rapidly activated upon contact with pro-inflammatory signals and together with infiltrating macrophages participate in CNS responses to infection, inflammation, injury, and neurodegeneration^12^. When pathologically insulted, either via endogenous or exogenous stimulations, microglia can transform to an “activated” state. Analogous to macrophages, activated microglia modify their shapes to enable their phagocytic functions and induce inflammatory response, releasing multiple cytokines and mediators in response to altered microenvironmental homeostasis. In turn, the actions of microglia critically determine the fate of other neural cells around^13^,^14^.

Despite the increasing reports studying the effects of autophagy in the CNS, little emphasis is placed on microglial cells. In this study, we investigated the effects of autophagy on the production of pro-inflammatory molecules in microglial cells treated with alpha-synuclein. We report that both trehalose and rapamycin activate autophagy in BV2 microglial cells and down-regulate the production of pro-inflammatory cytokines and nitric oxide (NO) in response to LPS and alpha-synuclein. This impacted on the effect of microglial activation on neuronal cells, leading to suppression of alpha-synuclein-induced neurotoxicity.

## Results

### Rapamycin and trehalose induce autophagy in BV2 microglial cells

**Figure 1:**
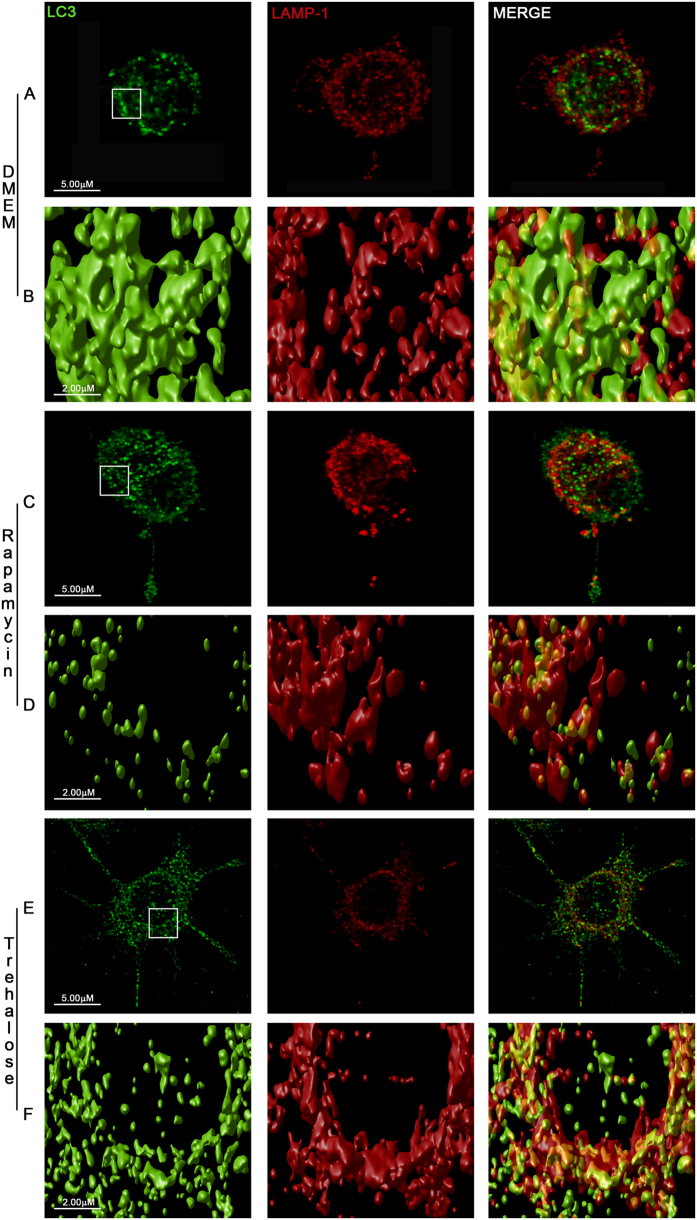
Autophagy induction in BV2 microglial cells. BV2 microglial cells were left untreated (**A**) or stimulated with rapamycin 100 nM (**C**) or trehalose 30 mM (**E**). After 24 h, cells were immunostained with anti-LC3B (green) and anti-Lamp-1 (red) antibodies. Images shown are z-stack projections. (**B,D and F**) are 3D surface-rendered magnifications of the selected area above. A typical LC3 puncta pattern is observed in BV2 stimulated cells (**D,F**). Merged images show fusion between autophagosomes and lysosomes (yellow).

**Figure 2:**
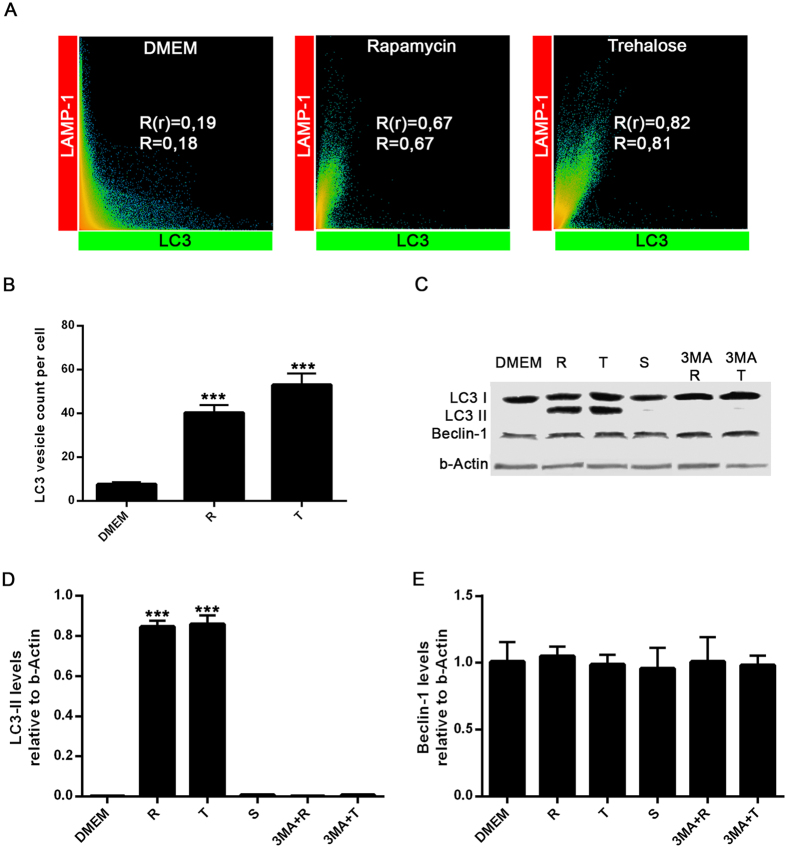
Evaluation of LC3-II and Beclin-1 levels in BV2 microglial cells. (**A**) Scatter plots represent colocalization analyses between LC3B and LAMP-1 using SVI Huygens Essential 14.1 software. Pearson coefficient (R) and Overlap coefficient (R[r]) are listed. (**B**) LC3 positive vesicles in unstimulated BV2 cells or treated with rapamycin (100 nM) or trehalose (30 mM) were determined using ImageJ particle counting plugin after cell deconvolution (n = 10). (**C**) BV2 cells incubated with 3-MA (2 mM; a specific inhibitor of autophagy) for 1 h at 37 °C were cultured in the presence or absence of rapamycin (100 nM) or trehalose (30 mM) for 24 h at 37 °C. Cells were lysed, and LC3B, Beclin-1 and β-Actin were examined by Western immunoblotting. Quantification of LC3-II (**D**) or Beclin-1 (**E**) from B relative to β-Actin by densitometry (one-way ANOVA followed by Post-Hoc Dunnet’s test; *n* = 3). Error bars represent SEM (*P < 0.05; **P < 0.01; ***P < 0.001).

We first examined the effects of classical inducers of autophagy on the formation of LC3B-labeled autophagosomes in the murine microglial cell line BV2. Morphometric analysis and enhanced visualization of autophagosomes by using 3D cell surface rendering approaches were performed after treatment of BV2 cells with trehalose and rapamycin. As expected, stimulation for 24 h with rapamycin (mTOR inhibitor), induced a typical LC3 puncta pattern in microglial cells (Figs 1 and 2A). Moreover, the LC3B expression colocalized with the late endosomal or lysosomal marker LAMP-1, indicating the fusion of autophagosomes with lysosomes (Fig. 1, Supplementary Videos 1–3). Similar results were obtained when BV2 cells were stimulated with trehalose, a molecule able to induce autophagy in a mTOR-independent manner (Fig. 1; Supplementary Videos 1–3). The autophagy marker LC3 was originally identified as a subunit of microtubule-associated proteins 1A and 1B (termed MAP1LC3)^15^. Soluble LC3 (Atg8) is called LC3B I, and detection of the autophagosome-specific form, LC3B II, is widely used to monitor autophagy^16^. We evaluated the conversion of LC3B I (nonlipidated form with lower electrophoretic mobility) to LC3B II (LC3 form C-terminally lipidated by phosphatidylethanolamine, displaying higher electrophoretic mobility) with immunoblots. Both, rapamycin and trehalose increased the intensity of the LC3B II relative to the intensity of β-actin band (Fig. 2B). The treatment of BV2 cells with sucrose failed to modify the levels of LC3B protein. When 3-Methyladenine (3-MA), a specific inhibitor of early stages of autophagy, was added to both rapamycin- and trehalose-stimulated microglial cell cultures, the intensity of LC3B II band decreased (Fig. 2B, compare lanes 5 and 6 with lanes 2 and 3, respectively), indicative of a reduced LC3B I to LC3B II conversion, consistent with the inhibition of autophagy induction. In addition, the levels of Beclin-1 were not affected by the treatment of microglial cells with rapamycin and trehalose, even in the presence of 3-MA (Fig. 2D). Overall, these results suggest that autophagy can be induced in microglial cells by either mTOR inhibitors or the action of trehalose, which acts in a mTOR independent manner.

### Autophagy down regulates the production of pro-inflammatory cytokines and nitric oxide production in BV2 microglial cells

**Figure 3:**
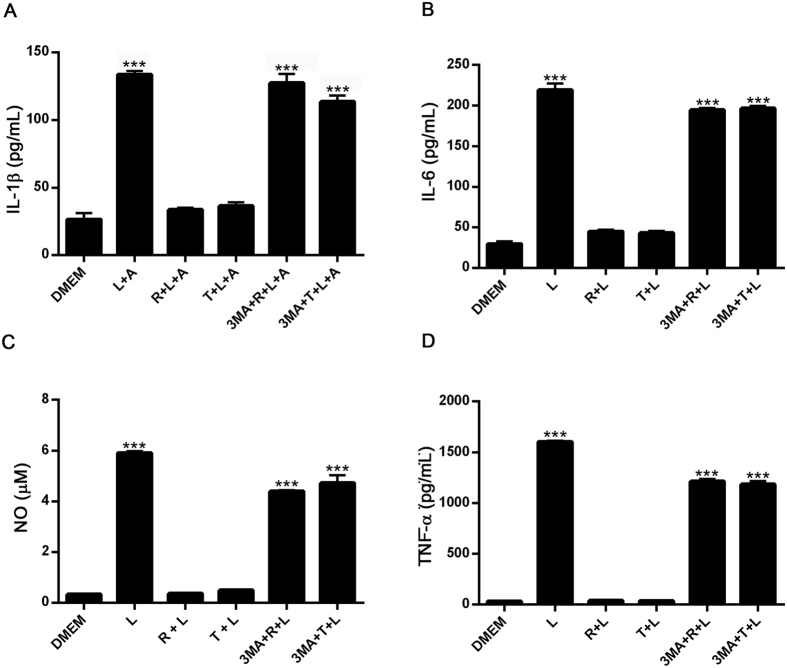
Effects of autophagy induction on IL-1β, IL-6, TNFα, and NO production in LPS-stimulated BV2 cells. BV2 cells incubated with 3-MA (2 mM) for 1 h at 37 °C were cultured in the presence or absence of rapamycin (100 nM) or trehalose (30 mM) for 24 h. After that, microglial cells were stimulated with LPS (0,5 ug/mL) for 24 h and the supernatants were isolated and analyzed by ELISA, for the measurement of IL-1β (**A**), IL-6 (**B**) and TNFα (**D**), respectively. For IL-1β determination, ATP (5 mM) was added for the last 3 h of LPS stimulation. NO levels (**C**) were determined by Griess assay. Results were analyzed by one-way ANOVA followed by Post-Hoc Dunnet’s test; *n* = 3. Error bars represent SEM (***P < 0.001). L = LPS, R = rapamycin, T = trehalose, A = ATP.

**Figure 4:**
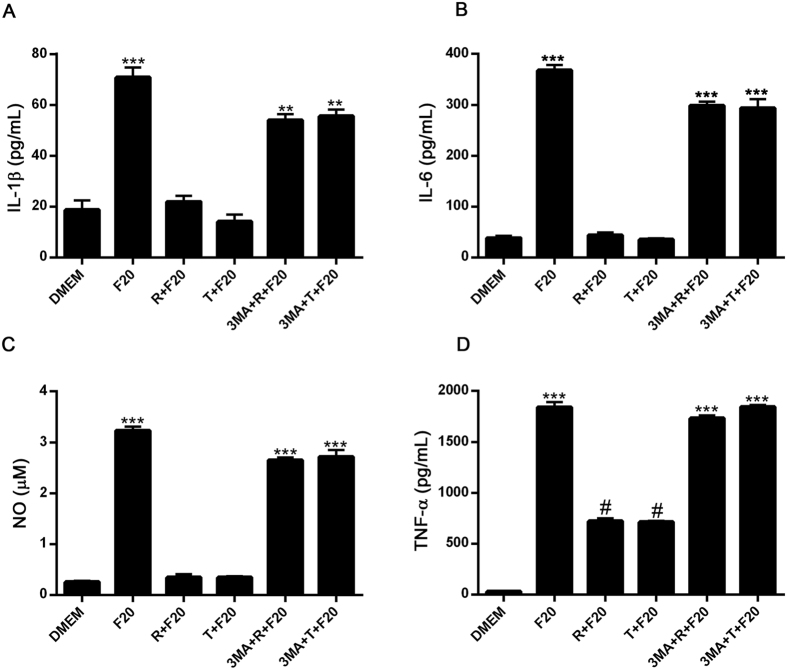
Effects of autophagy induction on IL-1β, IL-6, TNF-α, and NO production in alpha-synuclein-stimulated BV2 cells. BV2 cells incubated with 3-MA (2 mM) for 1 h at 37 °C were cultured in the presence or absence of rapamycin (100 nM) or trehalose (30 mM) for 24 h. After that, microglial cells were stimulated with alpha-synuclein fibers (20 uM) for 24 h and the supernatants were isolated and analyzed by ELISA, for the measurement of IL1-β (**A**), IL-6 (**B**) and TNFα (**D**), respectively. For IL-1β determination, ATP (5 mM) was added for the last 3 h of alpha-synuclein stimulation. NO levels (**C**) were determined by Griess assay. Results were analyzed by one-way ANOVA followed by Post-Hoc Dunnet’s test; *n* = 3. Error bars represent SEM (***P < 0.001). F20 = alpha-synuclein fibers (20 uM), R = rapamycin, T = trehalose.

We next examined the capacity of autophagy to modulate the production and secretion of pro-inflammatory mediators in LPS- or alpha-synuclein fibers-stimulated microglial cells. As shown in Fig. 3, after 24 h culture in the presence of LPS or alpha-synuclein fibers, BV2 microglial cells secreted increased levels of IL1-β, IL-6, NO and TNFα (Figs 3 and 4).

When we compared effects of monomeric vs. fibrilar alpha-synuclein on the stimulation of pro-inflammatory mediator production by microglial cells, we observed that alpha-synuclein fibers were more potent inducer of pro-inflammatory mediator production in microglial BV2 cells (Supplementary Figure S1). Later on, we observed that both, rapamycin and trehalose, down-regulated the production and secretion of pro-inflammatory mediators in BV2 cells treated with LPS and alpha-synuclein (Figs 3 and 4). In addition, when 3-MA, a specific inhibitor of early stages of autophagy, was added to microglial cells cultures, the effects of trehalose and rapamycin on LPS- or alpha-synuclein-stimulated production of pro-inflammatory mediators were reversed (Figs 3 and 4). In additional experiments we observed that both LPS and alpha-synuclein induced the production of IL-10, although LPS was more potent than alpha-synuclein in this effect (Supplementary Figure S2). As expected, 3-MA had no effect on IL-10 production by LPS and alpha-synuclein-stimulated BV2 cells. Later on we confirmed the effects of autophagy on pro-inflammatory molecules production in primary microglial cells. LPS induced the secretion of TNF-a and IL-10 in primary microglial cells. Rapamycin down-regulated the production and secretion of TNF-α, but not IL-10 in primary microglial cells treated with LPS (Supplementary Figure S3). In addition, alpha-synuclein induced the production of IL-12p70, IL-6 and NO in primary microglial cells and rapamycin inhibited both responses (Supplementary Figures S3 and S4). These results suggest that induction of autophagy in microglial cells negatively regulates pro-inflammatory responses after TLR or alpha-synuclein stimulation.

### Autophagy down regulates LPS- and alpha-synuclein-induced p38 and ERK1/2 phosphorylation in BV2

**Figure 5:**
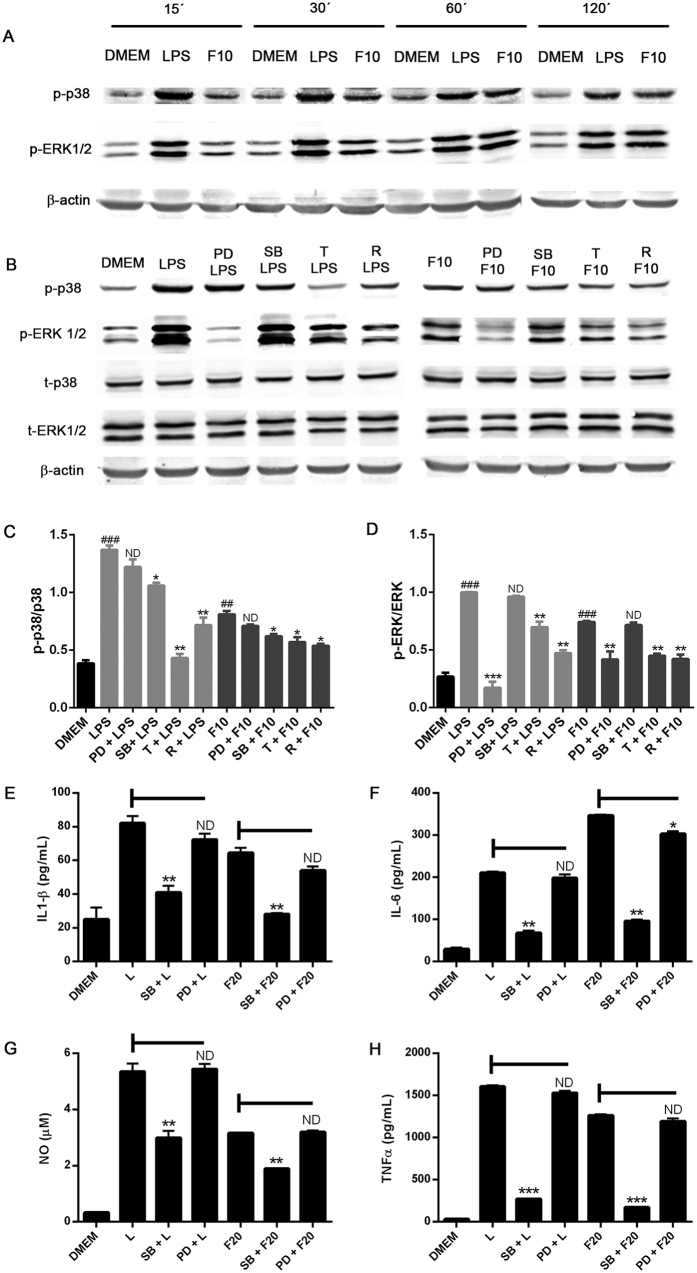
Autophagy modulation of p38 and ERK signalling in LPS and α-synuclein-stimulated BV2 microglial cells. (**A**) BV2 cells were stimulated with LPS (0,5 ug/mL) or alpha-synuclein fibers (10 uM) for 15 to 120 minutes. (**B**) BV2 cells were cultured in the presence or absence of PD98059 (50 uM) SB202190 (20 uM) for 1 h or treated with rapamycin (100 nm) or trehalose (30 mM) for 24 h. After that, microglial cells were stimulated with LPS (0,5 ug/mL) or alpha-synuclein fibers (10 uM) for 30 and 60 minutes, respectively. Cells were lysed and p38, p-p38, ERK1/2, p-ERK1/2 and b-actin levels were analysed by Western immunoblotting. Quantification by densitometry of p-p38 (**C**) or p-ERK1/2 (**D**) from B relative to p38 or ERK1/2, respectively. (One-way ANOVA followed by Post-Hoc Dunnet’s test; *n* = 3). Error bars represent SEM (*P < 0.05; **P < 0.01) (^###^P < 0.001 compared to DMEM). BV2 cells were cultured in the presence or absence of SB (20 uM) or PD (50 uM) for 1 h at 37 °C. After that, microglial cells were stimulated with LPS (0,5 ug/mL) or α-synuclein fibers (20 uM) for 24 h and the supernatants were isolated and analyzed by ELISA, for the measurement of IL-1β (**E**), IL-6 (**F**) and TNF-α (**H**), respectively. NO levels (**G**) were determined by Griess assay. Results were analyzed by one-way ANOVA followed by Post-Hoc Dunnet’s test; *n* = 3. Error bars represent SEM (***P < 0.001). L = LPS, R = rapamycin, T = trehalose, F10 = alpha-synuclein fibers 10 uM, F20 = α-synuclein fibers 20 uM.

The requirement of MAPKs, p38, and ERK1/2 in particular has been well documented for activation of microglial cells in response to pro-inflammatory molecules^12^,^17^. To elucidate the mechanistic basis for the effect of autophagy on LPS and alpha-synuclein signaling in microglial cells, we evaluated the capacity of trehalose and rapamycin to regulate MAPKs activation. Figure 5A shows that both LPS- and alpha-synuclein induced a rapid and persistent phosphorylation of p38 and ERK1/2 MAPKs in BV2 cells. Moreover, these effects were attenuated by specific inhibitors of these MAPKs pathways (SB202190 and PD98059, respectively)(Fig. 5B,C and D). However, only p38 inhibitor SB202190, but not MEK1/2 inhibitor PD98059, attenuated the effect of LPS or alpha-synuclein on the enhancement of production and secretion of IL1-β, IL-6, NO and TNFα (Fig. 5E–H). We therefore evaluated whether MAPKs might be potential targets for autophagy to disrupt the LPS- and alpha-synuclein-induced signaling cascade in microglial cells. Our results revealed that both rapamycin and trehalose were able to reduce the LPS- and alpha-synuclein-induced phosphorylation of p38 and ERK1/2 in BV2 cells (Fig. 5B,C and D). Thus, these results indicate that both LPS and alpha-synuclein induce activation of p38 and ERK1/2 in microglial cells and p38 activation is required for induction of pro-inflammatory cytokines and NO production. Moreover, the capacity of autophagy to down-regulate microglial cell activation is associated with the inhibition of LPS- and alpha-synuclein-stimulated MAPKs activation.

### Autophagy in microglial cells inhibits LPS- and alpha-synuclein-induced neuronal cell death

**Figure 6:**
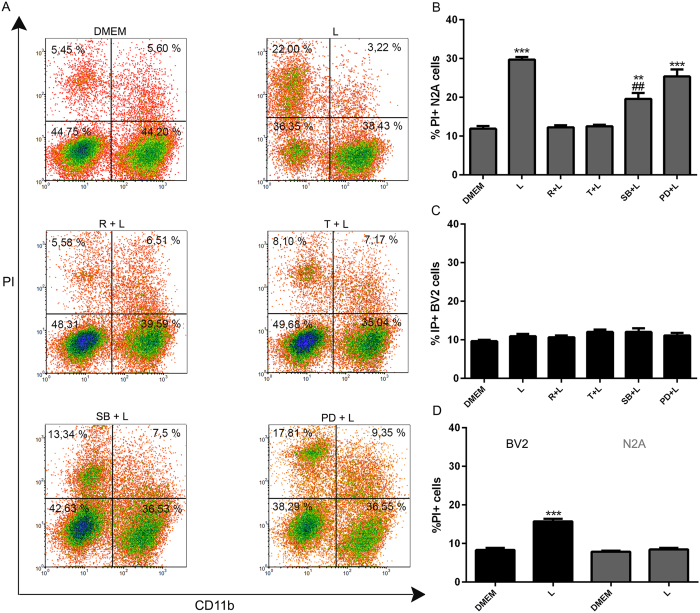
Modulation of LPS-induced neuronal cell death by inducing microglial autophagy. (**A**) BV2 and N2A cells were co-cultured in a 1:1 ratio and left untreated or stimulated with LPS for 48 h. Firstly, BV2 microglial cells were treated with rapamycin, trehalose, SB202190 (20 uM) or PD98059 (50 uM). After 24 h, BV2 cells were co-cultured with N2A cells and stimulated with LPS for 48 h. After that, cell death was evaluated using propidium iodide (PI) combined with anti-CD11b staining and subsequent flow cytometric analysis. (**B–D**) Percentages of neuronal CD11b−/IP+ and microglial CD11b+/IP+ cell death were determined and analyzed statistically by one-way ANOVA followed by Post-Hoc Dunnet’s test; *n* = 3. Error bars represent SEM (***P < 0.001, **P < 0.01); (^##^P < 0.01 compared to LPS) (L = LPS, R = rapamycin, T = trehalose). (**D**) BV2 cells alone or N2A alone treated with LPS.

In neurodegenerative diseases, reactive glia shift toward a pro-inflammatory phenotype and release cytokines as well as potentially neurotoxic substances including NO^18^. Aggregated proteins activate microglial cells and induce the production of factors, such as pro-inflammatory cytokines (e.g., TNFα, IL-1-β, IL-6) and NO, that promote neuronal death^8^. We therefore examined the effects of microglial autophagy on neuronal cell death in a co-culture system in response to LPS or alpha-synuclein. N2A neuronal cells and BV2 microglial cells were co-cultured in the presence or the absence of LPS or alpha-synuclein fibers and then neuronal and microglial cell (CD11b+) death were analyzed by flow cytometry. A dose response of alpha-synuclein-induced neuronal cell death was performed and the results showed that 20 uM was the concentration with significant N2A cell death without microglial cell death (Supplementary Figure S5). LPS increased the frequency of CD11b- PI+ (dead) N2A neuronal cells but the viability of microglial cells was not modified in co-cultures (Fig. 6A and C). In addition, LPS slightly increased microglial cell death when these cells were cultured alone (Fig. 6B), nevertheless, it had no effect on N2A cells when cultured alone (Fig. 6B). Both, trehalose and rapamycin efficiently blocked LPS-induced N2A cell death in co-cultures (Fig. 6A and C). The LPS effects on N2A cells were attenuated by the specific inhibitor of p38 MAPK SB202190, but not MEK1/2 inhibitor PD98059 (Fig. 6A and C). The stimulation of co-cultures with LPS alone or in the presence or the absence of trehalose, rapamycin or MAPKs inhibitors failed to affect microglial cell viability (Fig. 6D).

**Figure 7:**
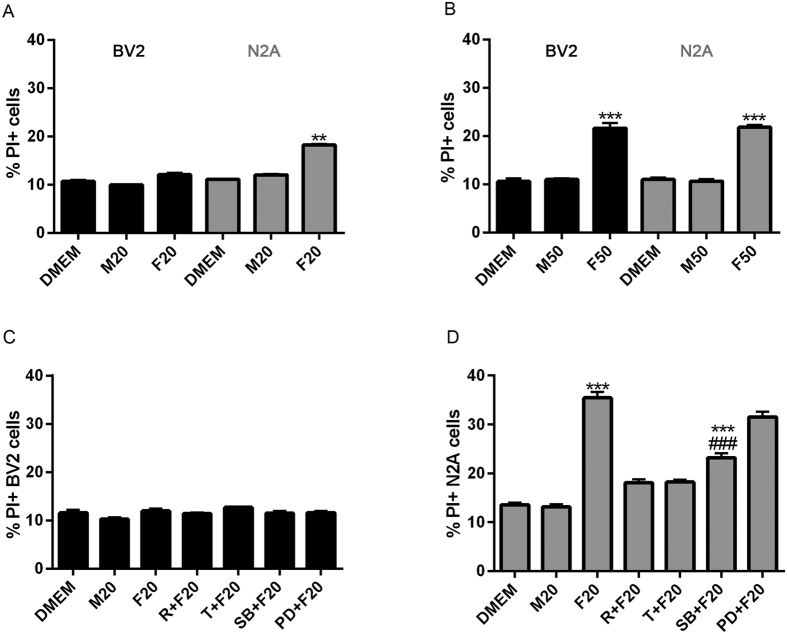
Effects of autophagy induction on alpha-synuclein-induced neuronal cell death. BV2 or N2A cells were stimulated with α-synuclein monomers or fibers for 48 h at 20 uM (**A**) or 50 uM (**B**) and cell death was determined using propidium iodide staining followed by flow cytometric analysis. BV2 and N2A cells were co-cultured in a 1:1 ratio and left untreated or stimulated with alpha-synuclein monomers or fibers for 48 h at 20 uM (**C,E**). The influence of microglial autophagy on neuronal cell survival was analyzed treating BV2 cells with rapamycin or trehalose before co-culture stimulation. After 48 h of stimulation, cell death in co-cultured cells was determined by flow cytometry. Results were analyzed by one-way ANOVA followed by Post-Hoc Dunnet’s test; *n* = 3. Error bars represent SEM (*P < 0.05; **P < 0.01; ***P < 0.001). M20 = α-synuclein monomers 20 uM, F20 = alpha-synuclein fibers 20 uM, M50 = alpha-synuclein monomers 50 uM, F50 = α-synuclein fibers 50 uM, R = rapamycin, T = trehalose.

Similar results were obtained when the co-cultures were stimulated with alpha-synuclein instead LPS, showing that alpha-synuclein fibers promote N2A neuronal cell death and this effect was blocked by trehalose and rapamycin and attenuated by p38 MAPK inhibitor SB202190 (Fig. 7D). These results suggest that induction autophagy in microglial cells results in the inhibition of microglial-associated neuronal cell death. Both, LPS and alpha-synuclein require p38 signaling to induce neuronal cell death.

**Figure 8:**
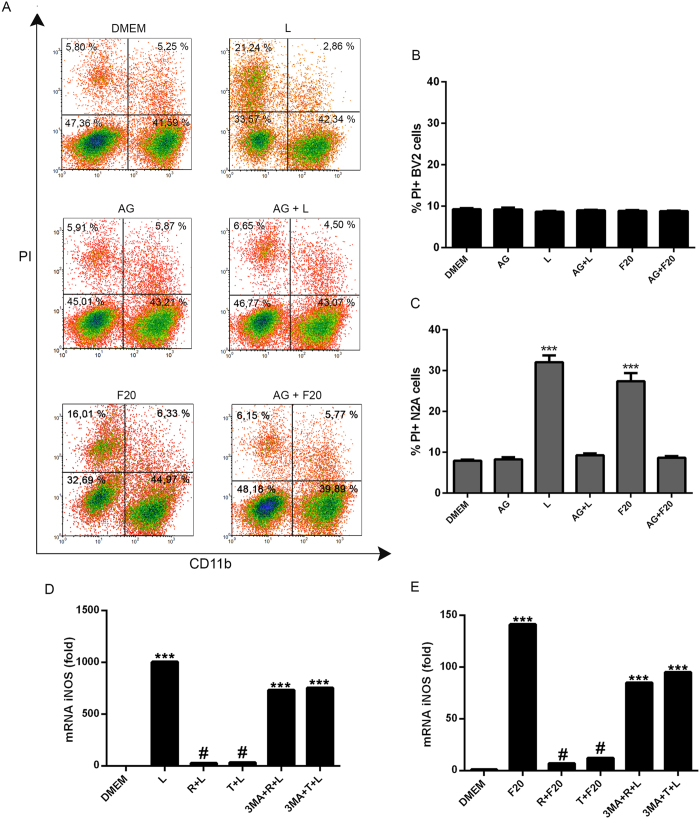
Participation of NO in LPS- and alpha-synuclein-induced neuronal cell death. (**A**) Microglial and neuronal co-cultures were stimulated with LPS or left untreated for 48 h. Co-cultures incubated with aminoguanidine (90 uM) for 1 h at 37 °C were cultured in the presence or the absence of LPS or alpha-synuclein fibers for 48 h and, respectively. After that, cell death was determined by flow cytometry. Percentages of microglial CD11b+/IP+ (**B**) and neuronal CD11b−/IP+ (**C**) cell death were determined and analyzed statistically by one-way ANOVA followed by Post-Hoc Dunnet’s test; *n* = 3. Error bars represent SEM (***P < 0.001). L = LPS, R = rapamycin, T = trehalose. BV2 cells incubated with 3-MA (2 mM) for 1 h at 37 °C were cultured in the presence or absence of rapamycin (100 nM) or trehalose (30 mM) for 24 h. After that, microglial cells were stimulated with LPS (**D**) or alpha-synuclein fibers (**E**) for 24 h. iNOS mRNA expression was calculated by qPCR using the comparative Ct method, relative to the housekeeping gene RPLP0 and the reference sample, DMEM (*n* = 3); data presented as fold change (2^−ΔΔCt^). Results were analyzed by one-way ANOVA followed by Post-Hoc Dunnet’s test. Error bars represent SEM (***P < 0.001). L = LPS, R = rapamycin, T = trehalose, F20 = alpha-synuclein fibers 20 uM.

LPS and alpha-synuclein were able to enhance the production of pro-inflammatory molecules, such as NO, in microglial cells, which in turn may induce death of neuronal cells. Therefore, we studied the involvement of NO in LPS or alpha-synuclein-induced neuronal cell death. First of all, aminoguanidine (AG), a specific inhibitor of nitric oxide synthase (iNOS), efficiently blocked NO production in BV2 cells in response to LPS or alpha-synuclein (Supplementary Figure S6). Conversely, the release of IL-1, IL-6, IL-10 and TNFα were no modified by AG treatment (Supplementary Figure S6). When co-cultured cells were treated with AG, we found inhibition of both LPS- or alpha-synuclein-induced neuronal cell death (Fig. 8A and C). In addition, we observed that both, LPS and alpha-synuclein increased the levels of iNOS gene expression in BV2 cells (Fig. 8D and E). These effects were inhibited by the treatment of BV2 cells with trehalose and rapamycin (Fig. 8D and E), however, when autophagy was inhibited in these cells by incubation with 3-MA, LPS- or alpha-synuclein-induced iNOS gene expression was restored (Fig. 8D and E). These results suggest that autophagy may regulate microglial-dependent neuronal cell death, through the modulation of the effector molecule NO.

## Discussion

Control of microglial cell activation is crucial for the regulation of the inflammatory responses and the host defense in the CNS. In this study, rapamycin and thehalose effectively induced autophagy in BV2 microglial cells and this response was associated with a reduction of pro-inflammatory molecules, including NO, in response to LPS and alpha-synuclein. Autophagy also modulated the phosphorylaytion of p38 MAPKs in BV2 cells, which was required for NO production. Therefore autophagy may act as a modulator of pro-inflammatory effects of TLR4 and alpha-synuclein stimulation of microglial cells. To our knowledge, this is the first demonstration that autophagy can modulate alpha-synuclein-induced pro-inflammatory mediators in microglial cells that may act as neurotoxins.

The current knowledge about the role of autophagy in the CNS is still patchy^19^. Autophagosomes accumulate in several brain disorders^20^,^21^ and autophagy seems to be essential for neuronal homeostasis, plasticity and protein quality control in neurons^22^,^23^. Most of the existing literature related to autophagy in the CNS focuses on neurons and little is known about the effects of the autophagic process and its regulation in microglial cells^24^. Proper activation of microglia can be beneficial to wound repair and microenvironment reconstruction, but excessive activation of microglia may aggravate the damage^25^,^26^. As a result, autophagy may be a critical mechanism in homeostasis, controlling the state of activation in microglia, and autophagy defects in response to nutrient deprivation may increase the degree of microglial activation and inflammation^14^.

Stimulation of COS-7 and human neuroblastoma cells SKN-SH with trehalose induced autophagy and enhanced the clearance of aggregate-prone proteins^27^. Studies have explored the use of pharmacologic mTOR inhibitors, such as rapamycin, which potently inhibit downstream mTOR signaling and thereby induce autophagy^28^,^29^,^30^. In our present study, we show that treatment of BV2 microglial cells with both trehalose (mTOR independent autophagy activator) and rapamycin (mTOR inhibitor) increased the number of LC3 positive vesicles, increased levels LC3B II and colocalization of LC3B puncta with lysosomal protein Lamp-1 indicating activation of autophagic flux.

Inflammatory factors secreted by microglia play an important role in focal ischemic stroke and other CNS diseases. The mammalian target of rapamycin (mTOR) pathway is a known regulator of immune responses, but the role mTORC1 signaling plays in neuroinflammation is not clear. Our studies showed that induction of autophagy with rapamycin or trehalose suppressed the production of pro-inflammatory cytokines and NO in microglial cells in response to LPS or alpha-synuclein. Autophagy was also shown to regulate IL1-β secretion by degradation of inflammasome. Inhibition of autophagy stimulated lipopolysaccharide (LPS)-induced inflammasome activation via NLRP3 inflammasome activation^31^,^32^ and induction of autophagy led to the degradation of pro-IL1-β, as well as decreased production of IL-1β *in vitro* and *in vivo*^14^,^31^. Recently, He *et al*. found that trehalose inhibited the generation of IL-1β, IL-6, TNFα and NO in microglia stimulated with LPS^9^. He *et al*., showed that BV2 microglial stimulation with the classical macrophage activator LPS could produce the release of cytokines and pro-inflammatory mediators, which in turn damage rat PC12 neurons through apoptosis effect. While trehalose could block the inflammatory response and further to inhibit the apoptosis of rat PC12 neurons from the cytotoxicity of activated BV2 cells^9^. We found that rapamycin (a mTOR-dependent autophagy inducer) and trehalose (a mTOR-independent autophagy enhancer) induce autophagic flux, and potently suppressed not only LPS-induced but also alpha-synuclein-induced production of IL-1β, IL-6, TNF-α and NO in BV2 cells. In addition, we observed that alpha-synuclein induced the production of IL-12p70 and IL-6 in primary microglial cells and rapamycin inhibited both responses. Moreover, we observed that alpha-synuclein fibers increased the production of NO in primary microglial cells and rapamycin suppressed this response. Our results are in agreement with a recent publication showing that rapamycin induced increased LC3+ puncta and LC3II levels^33^. Moreover, LPS seemed to increase LC3+ puncta but this response coud not be blocked by autophagy inhibitors wortmanin and an ATG5 siRNA. Finally, they show that rapamycin attenuated IL-6, iNOS mRNA, NO release and BV2 cell death, however, the induction of autophagic flux was not described^33^. In this manuscript we show that both rapamycin and trehalose induce autophagic flux, and potently suppressed not only LPS-induced but also alpha-synuclein-induced production and release of IL-1β, IL-6, TNF-α and NO in BV2 cells and TNF-α, IL-6, IL-12p70 and NO in primary microglial cells. Our results confirm that induction of autophagic flux in microglial cells leads to the suppression of pro-inflammatory molecules release in response to a TLR4 ligand and a PD-associated pathogenic protein.

Recently, Giegerich *et al*. showed that autophagy machinery participates in degradation of PELI3 and inhibition of pro-inflammatory IL-1β expression in macrophages activated by LPS^34^. The E3 ubiquitin ligase and scaffold protein PELI3 belongs to a new family of evolutionary conserved proteins in the TLR signaling cascade^34^,^35^. So far, PELI3 is implicated as activator of MAPK14/p38a, MAPK8/JNK1-MAPK9/JNK2, and MAPK1/ERK2-MAPK3/ERK1 signaling cascades of TLR/ IL1R pathways^34^. We examined whether trehalose and rapamycin have the capacity to regulate MAPKs activation induced by stimulation with LPS or alpha-synuclein. We found that both LPS and alpha-synuclein induce activation of p38 and ERK1/2 in microglial cells and p38 activation is required for induction of pro-inflammatory cytokines and NO production. In addition, autophagy was able to down-regulate LPS- and alpha-synuclein-stimulated MAPKs activation. In line with these results several reports indicate that MAPKs are target for modulation of neuroinflammation^12^,^17^,^36^,^37^.

Aggregated proteins, such as Amyloid beta and alpha-synuclein, and TLR-ligands activate microglial cells and can induce microglial and neuronal toxicity^11^,^38^,^39^. Activated microglia can cause neurotoxic effects, by accelerating the production of pro-inflammatory mediators, such as NO, PGE2, and pro-inflammatory cytokines IL-6, TNFα, and IL-1^9^. We found that both, LPS and alpha-synuclein require p38 signaling to induce neuronal cell death. Moreover, the induction of autophagy, that was capable to inhibit p38 phosporylation, resulted in the inhibition of microglial-associated neuronal cell death through the modulation of the effector molecule NO. While autophagy can eliminate aggregated proteins, defective mitochondria, and excessive reactive oxygen species (ROS) that could induce DNA damage and cell death, inadequate or defective autophagy promotes neuronal cell death in most of neurodegenerative disorders. It is becoming increasingly evident that the cell response may shift gradually from elimination of damaged proteins and organelles by autophagy, which leads to its recovery, to the induction of apoptotic or necrotic pathways determining cellular demise under neurodegenerative disorders^40^,^41^.

Although no solid evidence about the role of microglial autophagy on PD has emerged, previous research showed that dysregulation of autophagy is prominent at distinct stages in PD, where alpha-synuclein aggregates in ‘Lewy bodies’^42^. A newly revealed autophagy step that is disturbed in this disease is endosomal protein sorting to the Golgi, by mutating a retromer protein, VPS35, which then inhibits WASH complex trafficking to endosomes and ultimately autophagosome formation^43^. The autophagic machinery malfunction can extend its detrimental effects to neighbouring neurons, as it induces exocytosis and intercellular transfer of the aggregates^44^. Of note, disruption in mitophagy has emerged as a major cause in PD. In particular, mutated forms of PINK1 and PARKIN, which have been implicated in PD, are essential for mitochondrial biogenesis and recycling. Therefore, mitochondrial quality control by mitophagy is disrupted in this neurodegenerative disease^45^. In this manuscript, we show that alpha-synuclein fibers increased the production of NO in microglial cells and rapamycin suppressed this response. In addition, rapamycin and trehalose inhibited LPS- and alpha-synuclein-induced p38 and ERK1/2 phosphorylation in BV2 cells and microglial cell-induced neurotoxicity, which was dependent of p38 MAPK activation and NO production.

Autophagy may potentially regulate microglial cell function by different mechanisms and pathways. Researches focused in these topics are just started in last few years and further effort is required to dissect each pathological situation. In this sense, our manuscript shows novel results demonstrating that autophagy can modulate alpha-synuclein-induced pro-inflammatory mediators in microglial cells that may act as neurotoxins. Our manuscript provide novel and detailed data that associate the induction of autophagic flux using both mTOR-dependent and mTOR-independent stimuli, with the suppression of the pro-inflammatory mediator release in microglial cells activated with LPS and also with a PD-related pathogenic protein such as alpha-synuclein. In addition our paper provides evidences of molecules and pathways involved in the neurotoxic effect of microglial cells. We proposed NO as a main mediator driven neuronal cell toxicity and we showed that its microglial production could be negatively regulated by inducing autophagy. The interplay between microglial inflammatory response and microglial autophagy is inherent to acute CNS injury as well as the recovery stage or chronic neurodegeneration. Elucidating the mechanisms by which autophagy regulates the activation and inflammatory response in microglia both under physiological and pathological conditions should be beneficial for the design of therapeutic approaches to neurodegenerative diseases in which microglial cell activation plays an important role.

## Materials and Methods

### Reagents

DMEM was purchased from Life Technologies (Grand Island, NY, USA). Fetal bovine serum (FBS), penicillin, glutamine and streptomycin were obtained from GIBCO Laboratories (Grand Island, NY, USA). Rapamycin, SB202190, PD98059 and antibodies against Beclin-1, p-p38, p38, p-ERK, ERK and B-actin were purchased from Cell Signaling Technology (Beverly, MA, USA). Antibodie against LC3B, trehalose, ATP and 3-MA were purchased from Sigma-Aldrich (St. Louis, MO, USA). Antibody against CD11b was purchased from Becton Dickinson (San Jose, CA, USA).

### Cell culture

The murine microglial cell line BV2 was a kind gift from Dr. Dennis J. Selkoe (Harvard Medical School, Center for Neurological Diseases, Bringham and Women’s Hospital, Boston, MA, USA). The murine N2A neuronal cell line was purchased from American Type Culture Collection (ATCC) (Manasas, VA, USA). The cells were grown in DMEM supplemented with 10% heat-inactivated FCS, 2 mM glutamine and 100 ug/ml streptomycin and maintained at 37 °C and 5% CO_2._ Mammalian PI3KC3 was blocked by the addition of 3-methyladenine (3-MA) 2 mM for 1 h before stimulation. Autophagy was induced by using rapamycin at 100 nM for 24 h or trehalose at 30 mM for 24 h. Control assays with sucrose were done in same conditions. LPS was used at 0,5 ug/mL and alpha-synuclein fibers and monomers were used at 10 uM, 20 uM and 50 uM at indicated time points. The election of 20 uM of alpha-synuclein for most of the experiments (which is a commonly used dose in the literature) was because the microglial cell activation was significant without microglial cell death (Supplementary Figure S4). P38 and ERK1/2 MAPK were inhibited by treating BV2 cells with SB202190 at 20 uM or PD98059 at 50 uM for 1 h, respectively.

### Preparation of monomeric and aggregated alpha-synuclein

Monomeric alpha-synuclein stock solutions were prepared in PBS buffer and 0.02% sodium azide and centrifuged (14100 g, 30 min) before use in order to remove possible aggregates. After that, solutions were sterilized by filtration (22 um pore size). Protein concentration was determined by absorbance using an ε275 of 5600 M^−1^ · cm^−1^. Fibrillation was achieved by incubating 400 μM monomeric alpha-synuclein stock solutions at 70 °C and 800 rpm in a Thermomixer5436 (Eppendorf), conditions that lead to faster aggregation kinetics^46^. Fibril formation was monitored using the ThioT (thioflavin T) fluorescence assay. Fibrils were isolated by three consecutive cycles of centrifugation (14000 g, 30 min) and resuspended in PBS buffer. Protein concentrations in monomeric units were determined by the absorbance of aliquots incubated in 6 M guanidinium chloride at 25 °C for 24 h. Endotoxin levels were evaluated by Limulus Amebocyte Lysate (LAL) assay. Endotoxin content was lower than detection limit (<0,24 EU/mL).

### Transmission Electron Microscopy

Aliquots of alpha-synuclein (5 ul) were adsorbed onto Formvar-coated carbon grids (200 mesh), washed with Milli-Q water, and stained with 1% (w/v) uranyl acetate. The samples were imaged in a JEM-1200 Ex (Jeol) transmission electron microscope equipped with a GATAN camera, model 785. (Supplementary Figure S5).

### Cytokine and nitric oxide assays

Supernatants from microglial cells stimulated with LPS or alpha-synuclein fibers or monomers for 24 or 48 h, respectively, were assayed for IL-1β, IL-6 and TNF-α production by ELISA according to the manufacturer’s instructions (Mouse ELISA Kit, BD Biosciences). To determine IL-1β levels, ATP was used at 5 mM for 3 h after LPS or alpha- synuclein stimulation to activate inflammasome complex. Nitric oxide production was measured as nitrite using the Griess assay. Aminoguanidine (AG; Sigma) was used at 90 uM for 1 h prior to stimulation in order to inhibit NO production.

### Evaluation of cell death by flow cytometry

BV2 microglial cells and N2A neuronal cells were washed twice with PBS and incubated with propidium iodide (PI) for 2 minutes in 300 uL of FACS buffer. Stained cells were analyzed by flow cytometry on a FACSCanto II cytometer (Becton Dickinson) using FCS De Novo Software. When cell death was evaluated in co-culture samples, an anti CD11b immunolabeling was used before PI staining to discriminate microglial cells (CD11b+) from neuronal cells (CD11b−).

### Western immunoblotting

After treatment with rapamycin or trehalose, 2 × 10^6^ BV2 microglial cells were harvested by centrifugation at indicated time points. After washing with PBS, cells were lysed using sample buffer. Subsequently, cells lysates were sonicated and boiled. Western blotting of LC3B, Beclin-1, p-p38, p38, p-ERK, ERK and β-actin was performed according to the manufacturer’s instruction using specific polyclonal antibodies (Cell Signaling Technology). Briefly, proteins were electrophoresed on 12% SDS-PAGE gel under reducing conditions and transferred on to Immun-Blot PVDF Membrane (Bio-Rad, Hercules, CA, USA). The membranes were blocked with 5% nonfat milk and 0.1% Tween-20 in TBS for 2 h at room temperature and then were incubated with primary antibodies overnight at 4 °C. Then, membranes were incubated with a infrared fluorescent dye (IRDye, LI-COR Biosciences, Lincoln, Nebraska, USA) for 1 h and 30 min at RT and protein bands were detected with an Odyssey Infrared Imaging System (LI-COR Biosciences).

### Fluorescence confocal microscopy

Cells were grown on coverslips and fixed in absolute methanol for 10 min at 4 °C. The slides were then incubated with 5% albumin (Sigma) in PBS for 1 h to reduce nonspecific binding of antibodies. An anti-LC3B (Sigma) and anti- lysosomal associated membrane protein 1 (LAMP-1) was applied and further incubated for 1 h at room temperature. After 3 rinses with PBS, the slides were incubated with Alexa Fluor 488 or Alexa Fluor 546 secondary antibodies (Invitrogen) for 60 min and DAPI was used for nuclear staining. The slides were analyzed under a laser scanning confocal fluorescence microscope (Olympus FV1000; Olympus, Tokyo, Japan). Spatial deconvolution, 3D surface-rendered images and 3D surface-rendered movies were carried out with SVI Huygens Software. Quantification of LC3 vesicles was performed using ImageJ software (NIH).

### RNA Isolation and Quantitative Reverse Transcription and Polymerase Chain Reaction (qRT-PCR) Analysis

Total RNAs were isolated from cells by using Trizol reagent (Life Technologies, Rockville, MD, USA) according to the manufacturer’s protocol. Total RNA was quantified by spectrophotometry. One microgram of total RNA was converted to cDNA using the High-Capacity cDNA Reverse Transcription Kit (Life Technologies) and following manufacturer’s protocol. Real-time PCR was performed on an applied Biosystems StepOnePlus™ Real-Time PCR System (Applied Biosystems; Life Technologies, Carlsbad, CA, U.S.A.) using SYBR^®^ Green Real-Time PCR Master Mix (Life Technologies), and relative quantification (RQ) was calculated by using StepOne™ software V2.2.2, based on the equation RQ = 2^−ΔΔCt^, where Ct is the threshold cycle to detect fluorescence. Ct data were normalized to the internal standard RPLP0. Primer sequences were as follow: iNOS; Sense: GTT CTC AGC CCA ACA ATA CAA GA, Anti-sense: GTG GAC GGG TCG ATG TCA C; RPLP0: Sense: GGG CAT CAC CAC GAA AAT CTC, Anti-sense: CTG CCG TTG TCA AAC ACC T.

### Statistical analyses

The results were analysed using one-way analysis of variance (ANOVA) model, as indicated for every experiment. GraphPad Prism 6.0 was used to carry out the computations for all analyses. Results represent mean ± SEM of at least three experiments. Statistical significance was defined as p ≤ 0.05.

## Additional Information

**How to cite this article:** Bussi, C. *et al*. Autophagy down regulates pro-inflammatory mediators in BV2 microglial cells and rescues both LPS and alpha-synuclein induced neuronal cell death. *Sci. Rep.* **7**, 43153; doi: 10.1038/srep43153 (2017).

## Supplementary Material

Supplementary Material

Supplementary Video 1

Supplementary Video 2

Supplementary Video 3
