# Supplementary material for: Autophagy down regulates pro-inflammatory mediators in BV2 microglial cells and rescues both LPS and alpha-synuclein induced neuronal cell death
Source: Sci Rep. 2017 Mar 3;7:43153. doi: 10.1038/srep43153 (PMC5335665; doi:10.1038/srep43153)
Supplement: Supplementary Material [file srep43153-s1.pdf]

Supplementary Material:

Autophagy down regulates pro-inflammatory mediators in BV2 microglial cells and rescues both LPS and alpha-synuclein induced neuronal cell death

Claudio Bussi[1], Javier Maria Peralta Ramos[1], Emilia A. Gaviglio[1], Daniela S. Arroyo[1], Jose Ignacio Gallea[2], Ji Ming Wang[3], Maria Soledad Celej[2] and Pablo Iribarren[1,*].

1 Centro de Investigaciones en Bioquímica Clínica e Inmunología (CIBICI-CONICET). Departamento de Bioquímica Clínica, 2 Departamento de Química Biológica, Centro de Investigaciones en Química Biológica de Córdoba (CIQUIBIC, CONICET), Facultad de Ciencias Químicas, Universidad Nacional de Córdoba, Córdoba, Argentina. 3 Laboratory of Molecular Immunoregulation, Cancer and Inflammation Program, Center for Cancer Research, National Cancer Institute at Frederick, Frederick, Maryland 21702, USA.

*****Correspondence and requests for materials should be addressed to P.I. (email: piribarr@fcq.unc.edu.ar).

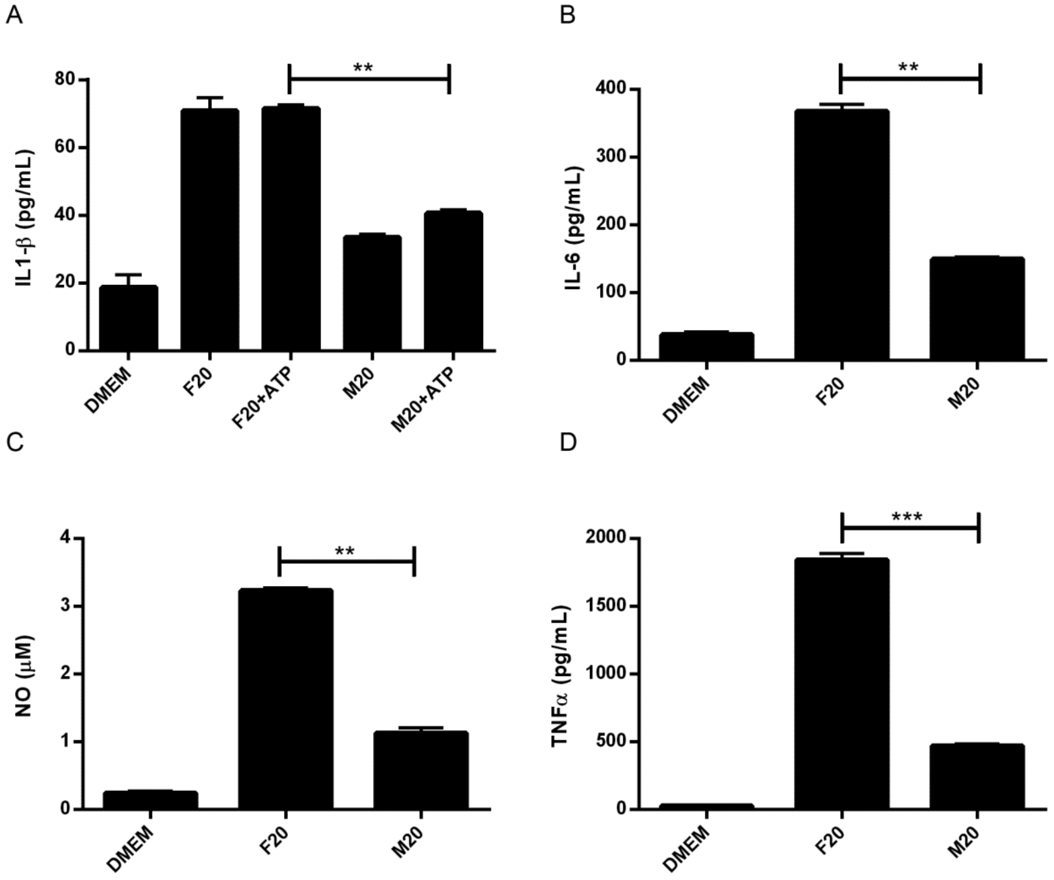

Figure S1. Comparison of pro-inflammatory mediators levels after monomeric or fibrilar alpha-synuclein stimulation in BV2 microglial cells. BV2 microglial cells were stimulated with alpha-synuclein fibers (20uM) or monomers (20uM) for 24h and the supernatants were isolated and analyzed by ELISA, for the measurement of IL-1β (A), IL-6 (B) and TNFα (D), respectively. NO levels (C) were determined by Griess assay. Results were analyzed by one-way ANOVA followed by Post-Hoc Dunnet´s test; *n* = 3. Error bars represent SEM (**, P < 0.01; ***, P < 0.001). M20= alpha-synuclein monomers 20uM, F20= alpha-synuclein fibers (20uM), A=ATP.

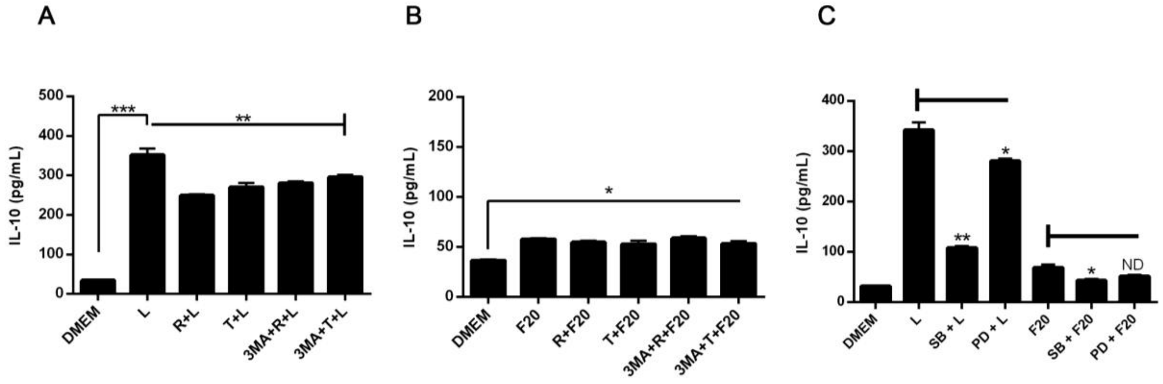

Figure S2. Effects of autophagy induction or MAPK inhibitors on IL-10 production in LPS or alpha-synuclein-stimulated BV2 cells. (A, B) BV2 cells incubated with 3-MA (2 mM) for 1 h at 37°C were cultured in the presence or absence of rapamycin (100nM) or trehalose (30mM) for 24 h. (C) Microglial cells were treated with SB (20uM) or PD (50uM) for 1 h at 37°C. After that, microglial cells were stimulated with LPS (0,5 ug/mL) (A) or alpha-synuclein fibers (20uM) (B) for 24h and the supernatants were isolated and analyzed by ELISA, for the measurement of IL-10. Results were analyzed by one-way ANOVA followed by Post-Hoc Dunnet´s test; *n* = 3. Error bars represent SEM (*, P < 0.05; **, P < 0.01; ***, P < 0.001). L=LPS, R=rapamycin, T=trehalose, F20= alpha-synuclein fibers 20uM.

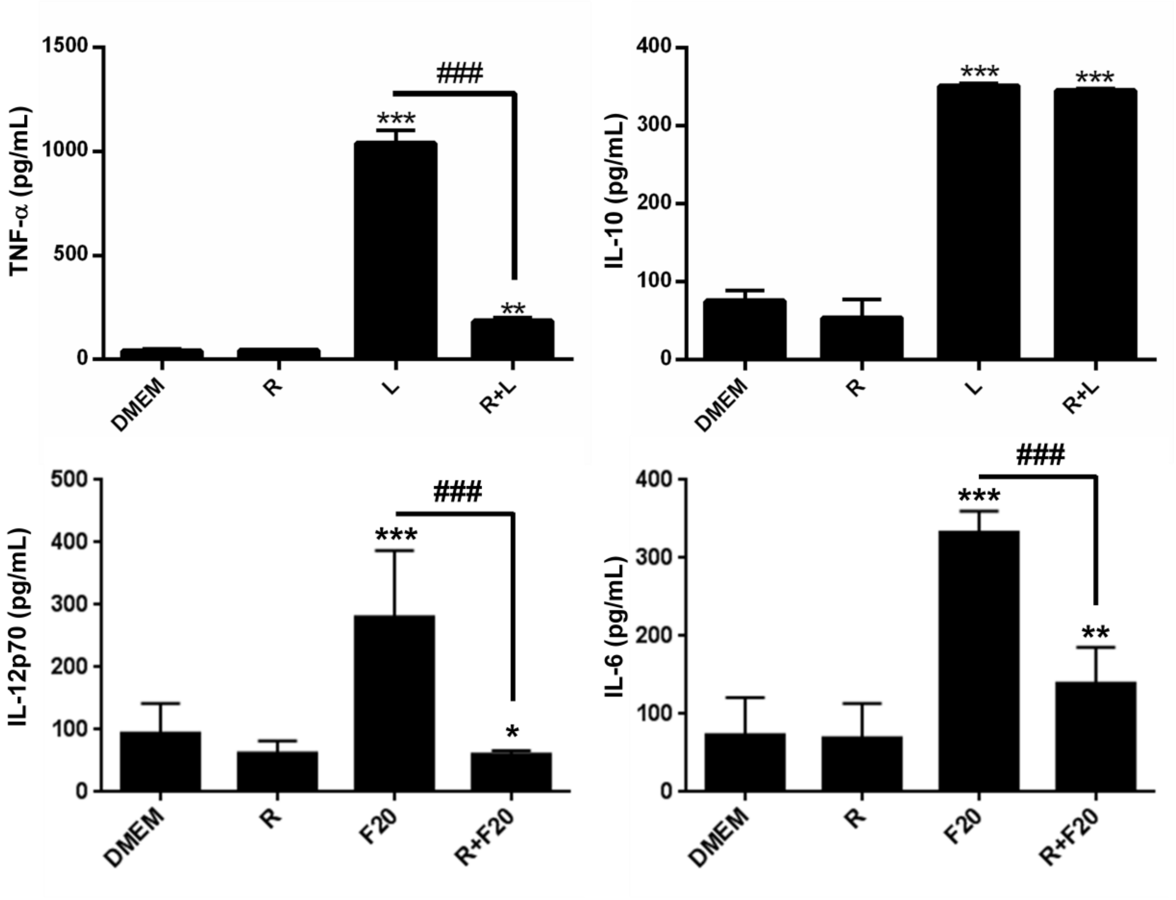

Figure S3. Effects of autophagy induction on TNFα, IL-10, IL-12p70 and IL-6 production in LPS- or alpha-synuclein-stimulated primary microglial cells. Primary microglial cells were cultured in the presence or absence of rapamycin (100nM) for 24 h. After that, microglial cells were stimulated with LPS (0,5 ug/mL) or alpha synuclein (20 uM) (F20) for 24h and the supernatants were isolated and analyzed by ELISA, for the measurement of TNFα and IL-10 or by flow cytometry using LEGENDplex™ Flow Assay Kit, Biolegend, for the measurement of IL-12p70 and IL-6. Results were analyzed by one-way ANOVA followed by Post-Hoc Dunnet´s test; *n* = 3. Error bars represent SEM (***, P < 0.001; **, P < 0.01; *, P < 0.05; ###, P < 0.01). L=LPS, R=rapamycin, F20=alpha-synuclein fibrils 20 uM.

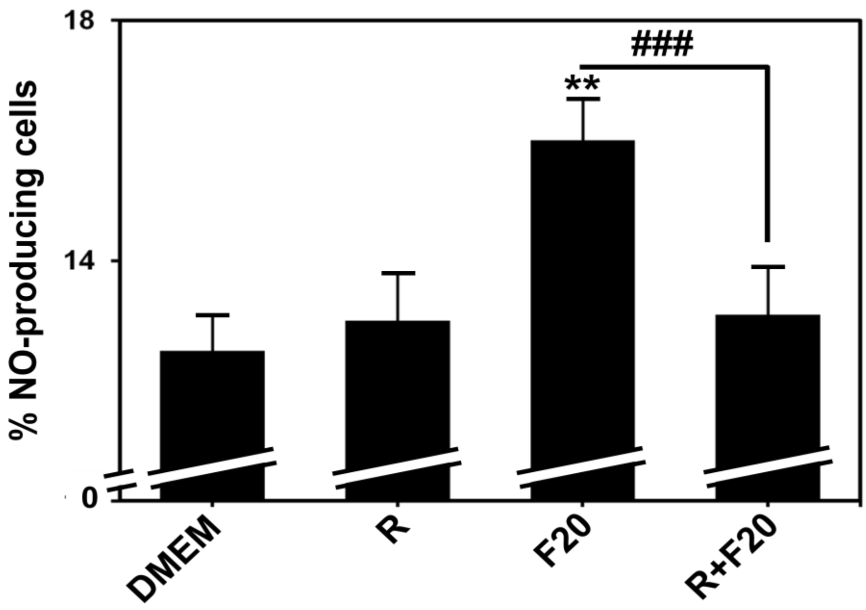

Figure S4. Effects of autophagy induction on NO production in alpha-synuclein-stimulated primary microglial cells. Primary microglial cells were cultured in the presence or absence of rapamycin (100nM) for 24 h. After that, microglial cells were stimulated with alpha synuclein (20 uM) (F20) for 24h and the supernatants were isolated and analyzed by flow cytometry using 4-Amino-5-Methylamino-2',7'-Difluorofluorescein Diacetate (DAF-FM, Molecular Probes), for the measurement of NO. Results were analyzed by one-way ANOVA followed by Post-Hoc Dunnet´s test; $n = 3$. Error bars represent SEM (**, $P < 0.01$). R=rapamycin, F20=alpha-synuclein fibrils 20 uM.

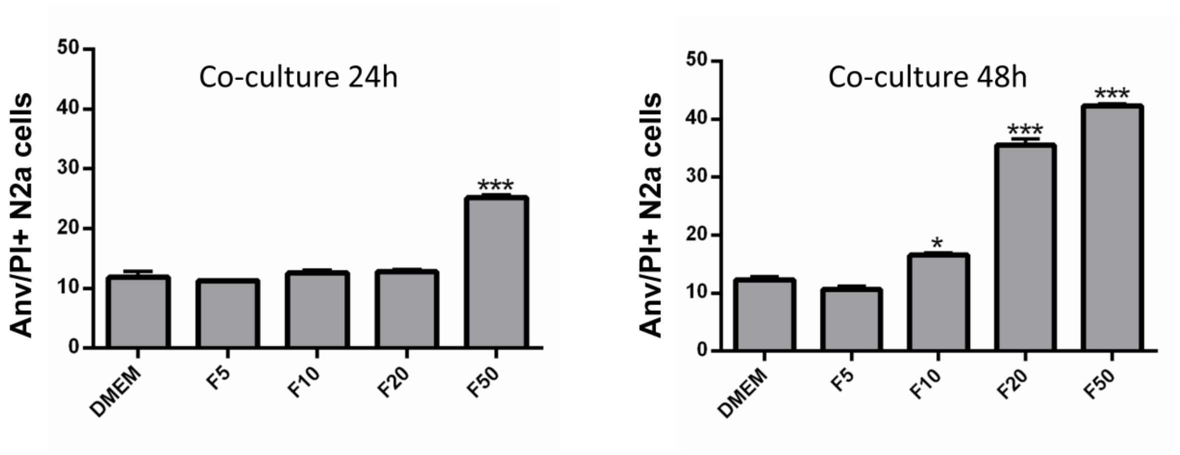

Figure S5. Dose response of alpha-synuclein-induced neuronal cell death. BV2 and N2A cells were co-cultured in a 1:1 ratio and left untreated or stimulated with alpha-synuclein fibers for 48h with concentrations from 5 to 50 uM. After 48h of stimulation, cell death in co-cultured cells stained with PI and Annexin V-FITC was determined by flow cytometry. Results were analyzed by one-way ANOVA followed by Post-Hoc Dunnet´s test; *n* = 3. Error bars represent SEM (*, $P < 0.05$; ***, $P < 0.001$). F5, F10, F20 and F50= alpha-synuclein fibers 5-50 uM, respectively.

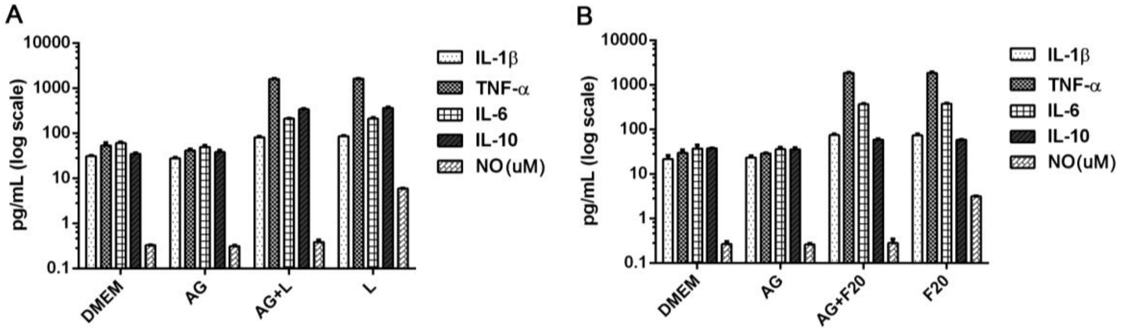

Figure S6. Aminoguanidine treatment only affects NO production in BV2 cells. BV2 cells incubated in the presence or absence of aminoguanidine (90 uM) for 1 h at 37°C were stimulated with LPS (0,5 ug/mL) (A) or alpha-synuclein fibers (20uM) (B) for 24h and the supernatants were isolated and analyzed by ELISA, for the measurement of IL-1β, IL-6 and TNF-α, respectively. NO levels were determined by Griess assay. Results were analyzed by one-way ANOVA followed by Post-Hoc Dunnet´s test; *n* = 3. Error bars represent SEM AG= aminoguanidine, L=LPS, F20= alpha-synuclein fibers (20uM)

A
B
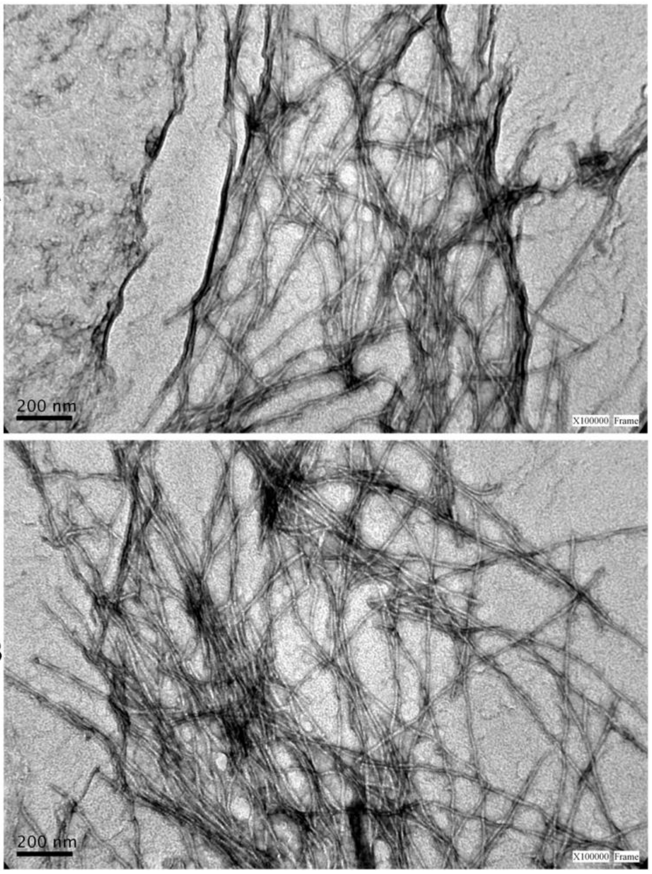

Figure S7. TEM of alpha-synuclein fibers.

Supplementary Videos 1-3. 3D Surface-rendered animations of untreated (1), Rapamycin (2) or Trehalose (3)-stimulated BV2 cells. LC3B and LAMP-1 are shown in green and red, respectively. Classical LC3 puncta pattern is observed in autophagy stimulated BV2 cells (2 and 3) in contrast with the diffuse distribution in untreated cells (1). 3D surface-rendered animations were done with SVI Huygens Essential 14.1 software by using deconvoluted z-stacks images.
